# The association of RBP4 with chronic kidney diseases in southern Chinese population

**DOI:** 10.3389/fendo.2024.1381060

**Published:** 2024-12-04

**Authors:** Tong Chen, Yu Liu, Shiquan Wu, Siyu Long, Ling Feng, Wenqian Lu, Wenya Chen, Guoai Hong, Li Zhou, Fang Wang, Yuechan Luo, Hequn Zou, Weihua Liu

**Affiliations:** ^1^ Department of Nephrology, South China Hospital of Shenzhen University, Shenzhen, China; ^2^ Guangdong Key Laboratory for Biomedical Measurements and Ultrasound Imaging, National Regional Key Technology Engineering Laboratory for Medical Ultrasound School of Biomedical Engineering, Shenzhen University Medical School, Shenzhen, China; ^3^ Department of Nephrology, The Third Affiliated Hospital, Southern Medical University, Guangzhou, China; ^4^ Department of Nephrology, Shenzhen Hospital, Southern Medical University, Shenzhen, China; ^5^ School of Medicine, The Chinese University of Hong Kong, Shenzhen, China; ^6^ Department of Nephrology, Shengli Clinical Medical College of Fujian Medical University, Fujian Provincial Hospital, Fujian, China

**Keywords:** retinol binding protein 4(RBP4), chronic kidney disease, insulin resistance, metabolic disorder, cross-sectional survey

## Abstract

**Background:**

Retinol binding protein 4 (RBP4), as a novel adipokine, has been proven to be highly related to insulin resistance, obesity, diabetes, hypertension, hyperuricemia and other metabolic diseases, which are all risk factors for chronic kidney disease (CKD). However, there is a lack of sufficient studies to explore the relationship between RBP4 and CKD, and no reports have described the predictive value of RBP4 for CKD. This study was designed to clarify the relationship between RBP4 and CKD and its potential predictive value.

**Methods:**

Our team has conducted a large-scale cross-sectional survey that contained 2117 individuals on the southern coast of China. Correlation test, logistic regression analysis were used to evaluate the association between RBP4 and CKD. Receiver operating characteristic (ROC) were used to evaluate the optimal cut-off and predictive value of RBP4 for predicting CKD.

**Results:**

By using the quartile grouping method, the population was divided into four groups according to the RBP4 level. As the RBP4 level increased, the prevalence of CKD also gradually increased among different groups. RBP4 was also correlated with various metabolic risk factors, such as blood glucose, blood lipids, blood pressure, waist circumference, uric acid, and with kidney function indicators such as creatinine, urine protein. Logistic regression analysis found that after adjusting for confounders, RBP4 remained significantly associated with CKD, independent of metabolic risk factors. ROC analysis showed that RBP4 as a single index, AUC (0.666) was superior to Scr, FBG, Log HOMA-IR, WC, TG, VLDL-C, UA, HDL-C, LDL-C, and that combining RBP4 indicator and other common risk factors of CKD can improve the accuracy for predicting CKD.

**Conclusion:**

This study found that the RBP4 was strongly correlated with CKD, RBP4 may become a valuable marker and have strong power for predicting CKD.

## 1 Introduction

Chronic kidney disease (CKD) causes gradual loss of kidney function. As CKD progresses, the biological functions of the kidney, such as filtering waste and excess fluid from the blood or maintaining the balance of minerals, hormones, and blood pressure, cannot be performed properly, eventually leading to various complications such as anaemia, bone disease, cardiovascular disease, and kidney failure (1). According to the National Health and Nutrition Examination Survey conducted from March 2017 to March 2020, the prevalence of CKD among American adults aged ≥18 years was 14%, and the prevalence of CKD showed an increase, reaching 38.4% in those aged 70 years and older (2). Besides, according to a 2018 cross-sectional study based on the China Health and Retirement Longitudinal Study, the prevalence of CKD among Chinese adults aged ≥45 years was 10.8%, affecting approximately 100 million adults (3). CKD is a major public health challenge worldwide, as it is associated with increased mortality, morbidity, and healthcare costs. Additionally, preventing or slowing CKD progression and its complications is possible by managing risk factors. Therefore, early detection and screening for CKD are necessary.

The glomerular filtration rate (GFR) is currently recognised as one of the best indicators of renal function; however, owing to its associated high cost, time-consuming factor, and invasiveness, its clinical application remains infrequent (4). The estimated glomerular filtration rate (eGFR) is one of the methods widely used as a substitute for the GFR in clinical practice. This was based on the results of blood tests that measured creatinine levels. However, creatinine levels can be affected by other factors such as diet, muscle mass, and other chronic illnesses; Cystatin C is a low-molecular-weight protein produced by all cells in the body and filtered by the kidneys. It serves as another biomarker for kidney function, with elevated levels indicating potential kidney impairment (5). However, BMI, diabetes, and inflammation may affect cystatin C levels independent of kidney function (6). therefore, eGFR or cystatin C alone is a poor predictor of future kidney disease and an insensitive marker of disease progression (7). Thus, a simple, low-cost, and convenient indicator which can help in improve the accuracy for predicting CKD is required.

Retinol-binding protein 4 (RBP4) is a member of the lipocalin family and the main transport protein of the hydrophobic molecule retinol (also known as vitamin A) in the blood circulation. RBP4 is most highly expressed in the liver, followed by significant expression in all adipose tissues. However, its mRNA is also present in other tissues, including the kidney, retinal pigment epithelium, testes, brain, lung, and choroid plexus. The liver’s highest expression correlates with its large retinoid stores, which contain about 80% of the body’s retinoids (8). In recent years, there has been increasing interest in RBP4, as many studies have found that RBP4 has additional functions. Increasing evidence suggests that RBP4 induces insulin resistance (IR) (9, 10) and is closely associated with diabetes mellitus (DM) (11, 12), obesity (13), metabolic syndrome (MS) (14, 15), hyperuricaemia (HUA) (16, 17), non-alcoholic fatty liver disease (NAFLD) (18, 19), hypertension (HTN) (20, 21), and cardiovascular diseases (CAD) (22). Moreover, some studies have shown that RBP4 elevation could cause adipose tissue inflammation by activating innate immunity (23). Obesity and obesity-induced chronic inflammation, IR, HTN, HUA, MS, and CAD are all risk factors for CKD. However, studies on the association between RBP4 and CKD are lacking. To the best of our knowledge, this is the first study to investigate the association between RBP4 and CKD in a large community population.

This large-scale study aimed to explore the association between RBP4 and CKD in the general population and evaluate whether RBP4 can predict CKD. Furthermore, in situations where GFR results are unavailable, abnormally elevated RBP4 levels may provide additional information about CKD progression.

## 2 Materials and methods

### 2.1 Study population

This study conducts a cross-sectional survey in Wanzhai community, Zhuhai City, on the southern coast of China, from December 2017 to March 2018. This community is an aging community with a high prevalence of non-communicable chronic diseases. The research team screens over 2,000 community residents using a multistage stratified cluster sampling method, which ensures the representativeness of the sample and reduces sampling error. First, we randomly sample two communities from Wanzhai Town; second, we randomly sample 500 families from the two sampled communities; third, we include and sample all the residents aged 18 to 75 from these families. We exclude participants with (1) missing clinical data, (2) severe liver or kidney damage, (3) acute cardiovascular diseases, or (4) malignancy. A total of 2117 individuals are involved in this research. We divide the population into two groups based on whether they have CKD: 1707 participants without CKD and 410 participants with CKD. The Ethics Committee of the Third Affiliated Hospital of Southern Medical University approves the epidemiological investigation and it complies with the Declaration of Helsinki. We obtain informed consent from all participants and address any doubts or questions by medical staff. After the participants agree and sign the informed consent, we collect and store the documents securely.

### 2.2 Data collection

We collected the socio-demographic data of the participants using a structured questionnaire, which included information on age, gender, current education, physical activity, current smoking, current alcohol consumption, history of hypertension, and history of diabetes. We also measured the anthropometric indices, including height, weight and waist circumference (WC) of the participants by trained researchers, and calculated their body mass index (BMI) [BMI= weight(kg)/height(m)2]. Moreover, we measured the systolic blood pressure (SBP) and diastolic blood pressure (DBP) of the participants in sitting position after 5 min of rest by a mercury desk-top sphygmomanometer. We repeated the measurement three times and calculated the mean blood pressure. Finally, we asked all participants to fast for at least 10 hours at night. The next morning, we collected fresh urine and venous blood samples from the participants using coagulation separation gel tubes (Shanghai Kehua, China). We processed the blood samples by gently inverting them three times to mix the blood and leaving them undisturbed for 20-30 minutes. Then, we centrifuged the blood samples at a speed of 3200-4000 rpm for 10 minutes. Subsequently, we sent all the samples (urine and blood) to the central laboratory of the Third Affiliated Hospital of Southern Medical University for examination. We measured the levels of low-density lipoprotein (LDL-C), high-density lipoprotein (HDL-C), and very low-density lipoprotein (VLDL-C) by a colorimetric method using a Roche cobas6000 apparatus (Penzberg, Germany), The level of high-sensitivity C-reactive protein(hs-CRP) was measured by immunotransmission turbidimetry. Serum creatinine (Scr), fasting blood glucose (FBG), and triglycerides (TG), blood urea nitrogen (BUN) and serum uric acid (UA) were measured by a standard enzymatic method. Fasting insulin was measured by an electrochemical luminescence method. The Homoeostatic Model Assessment of Insulin Resistance (HOMA-IR) was calculated using the formula: HOMA-IR = fasting blood glucose (mmol/L) x fasting insulin (mU/L)/22.5. Retinol binding protein 4 (RBP4) levels were measured using an immunoturbidimetric method (Shanghai Beijia Biochemical Reagent Company, China). Urinary albumin concentration was measured using immunoturbidimetric tests (Audit Diagnostics, Cork, Ireland), while urinary creatinine concentration was evaluated using Jaffe’s kinetic method (Audit Diagnostics, Cork). The urinary albumin to creatinine ratio (ACR) value was calculated based on the recorded concentrations of urinary albumin and urinary creatinine.

### 2.3 Definitions of HTN, DM, CKD, IR, MS and HUA

HTN: According to the National Institute for Health and Care Excellence (NICE), hypertension can be diagnosed by one of the following criteria: A clinic blood pressure reading of 140/90 mmHg or higher, or a history of hypertension.

DM: According to the American Diabetes Association (ADA), diabetes can be diagnosed by one of the following criteria: A fasting plasma glucose (FPG) level of 126 mg/dL (7.0 mmol/L) or higher, or a random plasma glucose of 200 mg/dL (11.1 mmol/L) or higher, or a history of diabetes.

The CKD was diagnosed by following criteria: eGFR <60 (mL/min/1.73m2) or ACR >30 mg/g. A formula from the Chinese-Modification of Diet Renal Disease (C-MDRD) study was used to calculate the eGFR: eGFR (mL/min/1.73 m2) = 175 × (Scr)−1.234× (Age)−0.179× (if female, ×0.79) (24).

IR is a condition where the body does not respond well to insulin, which is a hormone that regulates blood sugar levels. One way to measure IR is by using the HOMA-IR. Based on the review of previous epidemiological literature, this study establishes a cut-off point for defining IR as HOMA-IR > 2.69 mmol/L.mU/L (25).

HUA was defined as UA ≥420 mmol/L (7 mg/dL) in males and UA ≥360 µmol/L (6 mg/dL) in females (26).

The diagnostic criteria for MS are based on the global consensus jointly developed by the International Diabetes Federation (IDF), the American Heart Association (AHA), and the National Heart, Lung, and Blood Institute (NHLBI) in 2009 (27). The following five criteria were considered to diagnose MS: 1. abdominal obesity, defined as waist circumference ≥ 85 cm for men and ≥ 80 cm for women; 2. Abnormally elevated TG levels: TG ≥ 1.70 mmol/L; 3. elevated blood pressure: SBP ≥ 130 mmHg, DBP≥ 85 mmHg, or a history of hypertension; 4. Elevated fasting blood glucose: fasting blood glucose ≥ 5.6 mmol/L, or a history of diabetes; 5. Decreased HDL-C level: male HDL < 1.04 mmol/L, female HDL < 1.30 mmol/L. The subjects were diagnosed with MS when they met at least three of the five criteria.

### 2.4 Statistical analysis

Statistical Method: We used SPSS, version 20.0, for the statistical analysis. The data were divided into two types: numerical and categorical. Numerical data that followed a normal distribution were presented as mean ± standard deviation, and the t-test was used to compare the means between groups. Numerical data that did not follow a normal distribution were presented as median (25% quantile, 75% quantile), and the non-parametric rank sum test was used to compare the medians among groups. Categorical data were presented as absolute values (percentage), and the chi-square test was used to compare the proportions between groups. When the categorical data did not meet the assumptions for the chi-square test, Fisher’s exact test was used instead. Pearson’s correlation test was used to measure the correlation between numerical variables that followed a normal distribution. Spearman’ s correlation test was used to measure the correlation between numerical variables that did not follow a normal distribution. The strength of association was categorised as weak (r<0.3), moderate (0.3-0.6), strong (>0.6). Binomial logistic regression models were used to examine the relationship between CKD and RBP4. To enhance the association between this indicator and CKD, we divided the subjects into four subgroups based on quartiles of RBP4. The subjects in the first quartile were considered as the reference group in the binomial logistic regression analysis. A two-sided test with a significance level of 0.05 was used to determine statistical significance. A receiver operating characteristic (ROC) curve analysis was conducted to evaluate the predictive value of RBP4 for CKD. The analysis quantified the area under the ROC curve (AUC). The AUCs were compared to assess the performance of the indicators. Additionally, Youden’s index was calculated using the formula: Youden’s index = sensitivity + specificity - 1.

## 3 Results

### 3.1 Baseline characteristics of the study population

As shown in **Table 1**, this study included 2117 participants, 410 of whom had CKD. The mean age, percentage of physical activity, and histories of hypertension and diabetes were higher in CKD patients than in non-CKD group. In contrast, the education status was lower in CKD patients, and there were no significant differences in sex, smoking, and alcohol use between the two groups. In the comparison of physical and laboratory examinations, CKD group exhibited higher BMI, WC, SBP, DBP, FBG, TG, LDL-C, VLDL-C, insulin, HOMA-IR, Scr, BUN, UA, ACR, RBP4, and hs-CRP values and lower HDL-C levels and eGFR value than non-CKD group. Unexpectedly, this study did not find significant differences in the current smoking and drinking habits between the two groups. Furthermore, the CKD group had a higher percentage of physical activity than non-CKD group. Individuals might have changed their lifestyle after being diagnosed with CKD.

**Table 1:** Baseline characteristics of the study population.

Parameters	Non-CKD (1707)	CKD(410)	P
Sex, male (%)	610 (35.7%)	154 (37.5%)	0.489
Age (years)	53.89 (12.69)	65.05 (12.13)	<0.001
Education (high school or above, n (%))	641(40.4%)	93 (24.5%)	<0.001
Physical activity, n (%)	1008 (61.0%)	271 (67.2%)	0.021
Current smoking, n (%)	278 (16.7%)	74 (18.6%)	0.372
Current alcohol use, n (%)	81(4.9%)	22 (5.5%)	0.620
History of hypertension, n (%)	387 (22.7%)	227 (55.4%)	<0.001
History of diabetes, n (%)	114 (6.7%)	74 (18.0%)	<0.001
BMI (kg/m2 )	23.98 (3.37)	25.23 (3.65)	<0.001
Waist circumference (cm)	83.88 (9.80)	88.29 (9.73)	<0.001
Systolic blood pressure (mmHg)	131 (19)	146 (20)	<0.001
Diastolic blood pressure (mmHg)	82 (10)	86 (11)	<0.001
Fasting plasma glucose, mmol/L	5.16 (1.02)	5.88 (1.97)	<0.001
Triglyceride, mmol/L	1.53 (0.98)	1.89 (1.20)	<0.001
HDL-C, mmol/L	1.51 (0.34)	1.44 (0.36)	<0.001
LDL-C, mmol/L	3.18 (0.92)	3.35 (0.98)	<0.001
VLDL-C mmol/L	0.70 (0.44)	0.86 (0.54)	<0.001
Insulin (mU/mL)	8.85 (6.43-12.58)	11.12 (7.57-15.52)	<0.001
HOMA-IR (µU/mL ·mmol/mL)	1.99 (1.38-2.95)	2.71 (1.73-4.12)	<0.001
Serum creatinine (µmol/mL)	74.53 (14.60)	91.64 (42.72)	<0.001
Blood Urea Nitrogen (mmol/L)	5.07 (1.22)	6.12 (2.34)	<0.001
eGFR (mL/min/1.73 m2)	89.04 (14.38)	70.97 (20.99)	<0.001
ACR (mg/g)	9.29 (6.37-14.15)	45.00 (31.23-88.36)	<0.001
Serum uric acid (µmol/L)	341.68 (83.61)	383.08 (99.40)	<0.001
RBP4 (mg/L)	55.03 (14.99)	64.67 (18.47)	<0.001
hypersensitive C-reactive protein, mmol/L	1.26 (0.45-2.34)	1.89 (0.95-3.42)	<0.001

Data are shown as mean±standard deviation, median (interquartile range), or frequency (percentage).

BMI,body mass index; HOMA-IR, homeostasis model assessment of insulin resistance; eGFR, estimated glomerular filtration rate; RBP4, retinol-binding protein 4.

### 3.2 Clinical characteristics grouped by RBP4 interquartile range in different groups

**Figure 1:**
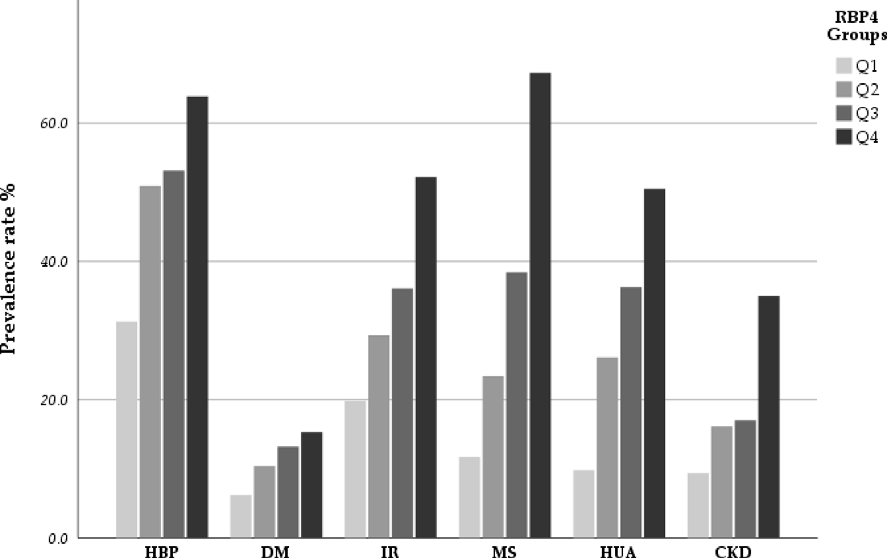
The incidence rate of HBP, DM, IR, MS, HUA and CKD stratified by quartiles of RBP4.The results indicated that there was a positive association between RBP4 with the risk of metabolic diseases and CKD in participants. As the RBP4 increased, the prevalence of these diseases also increased.

Based on the quartile method, the patients were divided into four groups according to their RBP4 levels. As shown in **Table 2** and **Figure 1**, the prevalence rates of HTN, DM, IR, MS, HUA, and CKD differed significantly between the groups. These results indicated a positive association between RBP4 and the risk of metabolic diseases. Participants in the highest RBP4 quartile had a significantly higher prevalence of HTN, DM, IR, MS, HUA, and CKD than those in the lowest quartile (P<0.001). In terms of the anthropometric indicators, BMI, WC, and BP differed significantly between the groups (P<0.05). Participants in the higher RBP4 quartile tended to have higher BMI, WC, and BP values. In contrast, the FBG, TG, HDL-C, HOMA-IR, Scr, BUN, eGFR, UA, and hs-CRP values were higher and HDL and eGFR values were lower in the fourth quartile (Q4) than in the first quartile (Q1).

**Table 2:** Clinical characteristics of study population grouped by RBP4 interquartile range.

	Study population (2117)				
Characteristic	RBP4(Q1)	RBP4(Q2)	RBP4(Q3)	RBP4(Q4)	P
Age (years)	50.92 (14.25)	55.85 (13.39)	58.12 (12.34)	59.32 (11.67)	<0.001
Prevalence of hypertension (%)	166 (31.3%)	269 (50.9%)	281 (53.1%)	338 (63.9%)	<0.001
Prevalence of diabetes (%)	33 (6.2%)	55 (10.4%)	70 (13.2%)	81 (15.3%)	<0.001
Prevalence of insulin resistance (%)	105 (19.8%)	155 (29.3%)	191 (36.1%)	276 (52.2%)	<0.001
Prevalence of metabolic syndrome (%)	62 (11.7%)	124 (23.4%)	203 (38.4%)	356 (67.3%)	<0.001
Prevalence of hyperuricemia (%)	52 (9.8%)	138 (26.1%)	192 (36.3%)	267 (50.5%)	<0.001
Prevalence of chronic kidney diseases(%)	50 (9.4%)	85 (16.1%)	90 (17.0%)	185 (35.0%)	<0.001
physical examination					
SBP (mmHg)	126 (19)	135 (20)	136 (19)	141 (18)	<0.05
DBP (mmHg)	78 (10)	83 (10)	83 (10)	86 (11)	<0.05
BMI (kg/m2)	22.87 (3.27)	23.93 (3.35)	24.72 (3.49)	25.37 (3.23)	<0.001
WC (cm)	79.42 (9.66)	83.85 (9.29)	86.88 (9.46)	88.79 (8.74)	<0.001
laboratory examination					
FPG (mmol/L)	5.00 (1.00)	5.21 (1.17)	5.39 (1.25)	5.60 (1.62)	<0.001
TG (mmol/L)	1.00 (0.34)	1.25 (0.42)	1.51 (0.58)	2.64 (1.44)	<0.001
HDL-C(mmol/L)	1.59 (0.33)	1.54 (0.32)	1.52 (0.35)	1.35 (0.33)	<0.001
HOMA-IR (mmol/L.mU/L)	1.65 (1.21-1.65)	1.95 (1.44-2.88)	2.18 (1.48-3.32)	2.78 (1.90-3.93)	<0.001
Serum creatinine (µmol/mL)	69.58 (13.40)	74.79 (16.35)	80.25 (19.20)	86.77 (36.15)	<0.001
Blood Urea Nitrogen (mmol/L)	4.77 (1.27)	5.16 (1.21)	5.48 (1.37)	5.69 (2.07)	<0.001
eGFR (mL/min/1.73 m2)	93.89 (15.59)	87.53 (16.40)	82.39 (15.41)	78.33 (18.10)	<0.001
Serum uric acid (µmol/L)	299.27 (71.07)	338.97 (76.95)	364.67 (84.04)	395.97 (90.72)	<0.001
hs-CRP (mg/L)	1.00 (0-1.92)	1.27 (0.54-2.44)	1.51 (0.70-2.75)	1.70 (0.91-2.86)	<0.001

### 3.3 Correlation between RBP4 and metabolic risk factors

**Table 3** presents the correlations between RBP4 and metabolic risk factors using Pearson’s or Spearman’s correlation analysis as the analytical method. Our results showed that RBP4 expression was positively associated with metabolic risk factors. RBP4 demonstrated a strong correlation with TG (r=0.696) and VLDL-C (r=0.696) and a moderate correlation with WC, HOMA-IR, Scr, eGFR, ACR, and UA (r>0.3). BP, FBG, HDL-C, LDL-C, insulin, BUN, and hs-CRP levels weakly correlated with RBP4 (r<0.3). Hence, TG, VLDL-C, WC, HOMA-IR, and UA levels are the key indicators of obesity, insulin resistance, lipid metabolism disorders, and hyperuricaemia. RBP4, a novel adipokine, was strongly or moderately correlated with these indicators. Scr, BUN, ACR and eGFR are indicators of kidney function. The correlation between RBP4 and these indicators suggests that RBP4 may also have the potential to be one of the indicators of kidney function.

**Table 3:** Correlation between RBP4 with metabolic risk factors.

Risk factors	RBP4	
	r	P
Systolic blood pressure (mmHg)	0.258	<0.001
Diastolic blood pressure (mmHg)	0.261	<0.001
Waist circumference	0.320	<0.001
Triglyceride, mmol/L	0.696	<0.001
Fasting plasma glucose, mmol/L	0.166	<0.001
HDL-C, mmol/L	-0.288	<0.001
LDL-C, mmol/L	0.075	<0.001
VLDL-C mmol/L	0.696	<0.001
Insulin (mU/mL)	0.268	<0.001
HOMA-IR (µU/mL ·mmol/mL)	0.306	<0.001
Serum creatinine (µmol/mL)	0.338	<0.001
eGFR (mL/min/1.73 m2)	-0.334	<0.001
ACR (mg/g)	0.301	<0.001
Serum uric acid (µmol/L)	0.402	<0.001
Blood Urea Nitrogen (mmol/L)	0.275	<0.001
hypersensitive C-reactive protein, mmol/L	0.189	<0.001

### 3.4 Association between RBP4 and presence of CKD

Binary logistic regression models were used to analyse the association between the RBP4 quartiles and CKD (**Table 4**). After adjusting for age, sex, education, activity, alcohol use, smoking, hypertension history, and diabetes history (Model 1), participants in the 4th quartile of RBP4 had a 3.467-fold increased risk of CKD compared to those in the 1st quartile (P<0.001). While further controlling for WC, BP, FBG, TG, HDL-C, LDL-C, and VLDL-C, the highest quartile of RBP4 still had a significantly higher risk of developing CKD (OR: 3.108, 95% CI, 1.932-4.999) compared to 1st quartiles. Furthermore, after controlling for UA, log hs-CRP, HOMA-IR, and eGFR (model 3), RBP4 still had significant ORs for the presence of CKD (OR: 1.862, 95% CI, 1.057-3.279). These results suggest that the association between RBP4 and CKD was independent of all risk factors mentioned above.

**Table 4:** Binary logistic regression analysis showing independent association between RBP4 and CKD.

	Model 1		Model 2		Model 3	
	OR(95%CI)	P	OR(95%CI)	P	OR(95%CI)	P
TyG						
Q1(Ref) Q2 Q3 Q4 P for trend	1 1.217(0.789-1.878) 1.320(0.864-2.017) 3.467(2.330-5.160)	<0.001 <0.001 <0.001 <0.001	1 1.118(0.718-1.742) 1.150(0.736-1.799) 3.108(1.932-4.999)	0.028 0.009 <0.001 <0.001	1 1.071(0.643-1.785) 1.006(0.603-1.679) 1.862(1.057-3.279)	0.791 0.983 0.031 0.034

Model 1 adjusted for age, gender, education, activity, alcohol use, smoking, hypertension history, diabetes history.

Model 2 adjusted for model 1 covariates plus WC, BP, FBG, TG, HDL-C, LDL-C, VLDL-C.

Model 3: adjusted for model 2 covariates plus Serum uric acid, log hs-CRP, HOMA-IR, eGFR.

### 3.5 Comparison of the predictive ability of RBP4 for CKD

**Figure 2:**
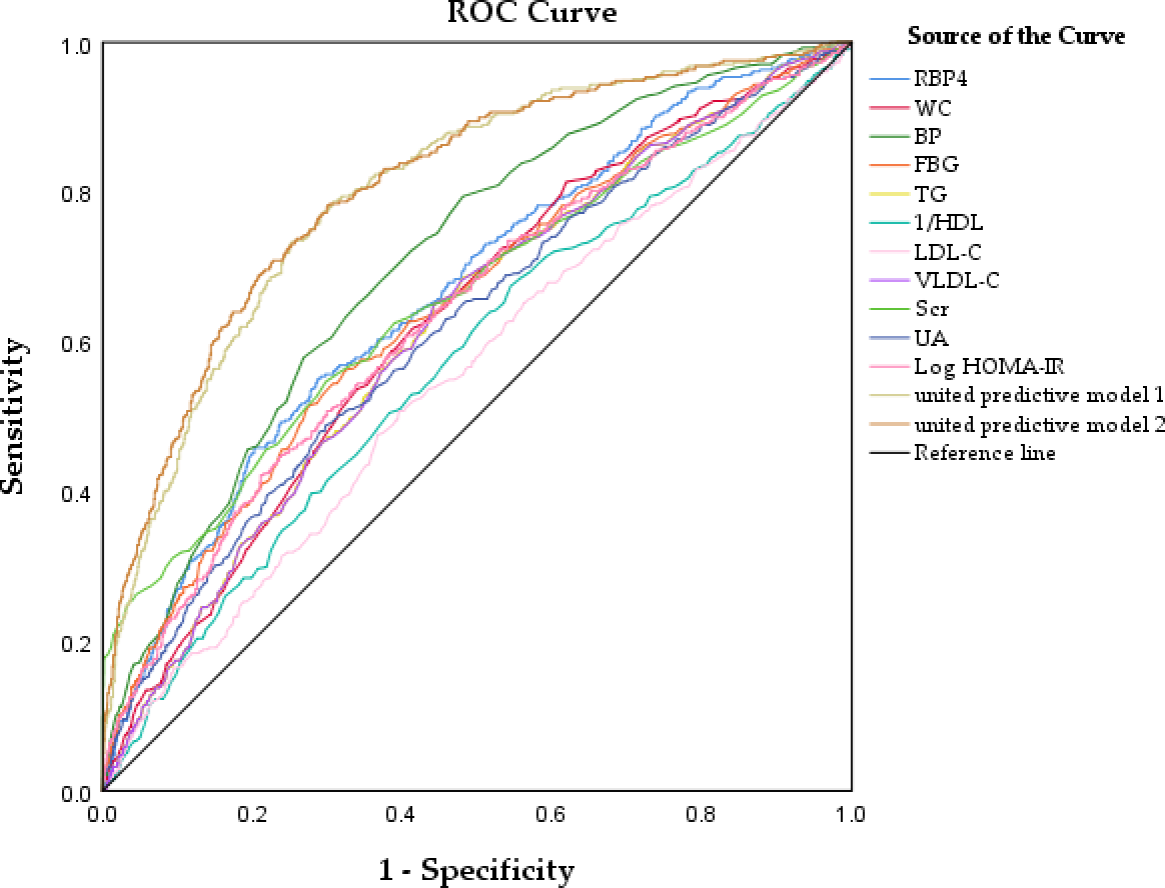
Receiver operator characteristic curves analysis of the value of RBP4 and other metabolic risk factors, united predicted model for predicting CKD.

This study assessed the diagnostic value of RBP4, BP, FBG, log HOMA-IR, WC, TG, VLDL-C, UA, Scr, HDL-C, and LDL-C levels in CKD using ROC curve analysis and comparison. **Table 5** and **Figure 2** present the results of this analysis. We also used a reciprocal approach to obtain HDL-C levels with the same tendency as other markers in the ROC curve analysis. The highest AUC was observed for BP (0.709) followed by RBP4 (0.666). RBP4 as a single index, with an AUC (0.666) was superior to Scr, FBG, Log HOMA-IR, WC, TG, VLDL-C, UA, HDL-C, and LDL-C.

**Table 5:** The Areas Under ROC Curve (AUC), sensitivity and specificity by the optimized cut-off points for RBP4 and other metabolic risk indicators in Predicting CKD.

Characteristic	AUC	Sensitivity	Specificity	Youden index	Cut-off	P
Blood pressure	0.709(0.682-0.736)	0.794	0.519	0.313	130	<0.001
RBP4	0.666(0.636-0.695)	0.551	0.711	0.262	60.5	<0.001
Serum creatinine	0.658(0.626-0.690)	0.549	0.702	0.251	81.5	<0.001
Fasting plasma glucose	0.647(0.617-0.678)	0.515	0.723	0.238	5.3	<0.001
Log HOMA-IR	0.637(0.607-0.668)	0.420	0.787	0.207	0.5	<0.001
Waist circumference	0.630(0.600-0.659	0.620	0.505	0.205	85.7	<0.001
Triglyceride	0.622(0.592-0.651)	0.649	0.557	0.206	1.3	<0.001
VLDL-C	0.621(0.590-0.651)	0.649	0.556	0.205	0.6	<0.001
Uric acid	0.621(0.690-0.652)	0.488	0.704	0.192	380.5	<0.001
1/HDL	0.573(0.541-0.605)	0.676	0.455	0.131	0.65	<0.001
LDL-C	0.554(0.522-0.586)	0.510	0.604	0.114	3.3	<0.001
United predictive model 1	0.801(0.777-0.825)	0.733	0.748	0.481	0.21	<0.001
United predictive model 2	0.810(0.786-0.834)	0.782	0.702	0.484	0.17	<0.001

United predictive model 1 includes age, gender, WC, BP, FBG, TG, HDL-C, LDL-C, VLDL-C, Log HOMA-IR, UA.

United predictive model 2 includes model 1 covariates plus RBP4.

The united model 1 included common risk variables related to CKD (age, sex, WC, BP, FBG, TG, HDL-C, LDL-C, VLDL-C, Log HOMA-IR, UA). The united model 2, which included RBP4, was similar to model 1. The ability of the united model 2 to predict CKD (AUC: 0.810) was stronger than that of model 1 (AUC: 0.801), suggesting that incorporating RBP4 with traditional risk indicators in the model yielded better results than including each common traditional risk indicator individually. Furthermore, the diagnostic value of a single indicator was limited. These results suggest that combining RBP4 indicators and other common risk factors for CKD can improve the accuracy of CKD prediction.

## 4 Discussion

Our results led to three major findings. Firstly, serum RBP4 levels were significantly higher in the CKD population than in the non-CKD population. As the serum RBP4 levels increased, the prevalence of CKD gradually increased from 9.4% in quartile 1 to 35% in quartile 4. The relationship between RBP4 and CKD prevalence may be related to metabolic risk factors, as the correlation analysis revealed that RBP4 positively correlated with WC, TG, HOMA-IR, UA, Scr, eGFR, and ACR (r>0.3). This finding is in accordance with those of previous studies which showed that RBP4 is significantly positively associated with IR, diabetes, and other metabolic diseases. Secondly, logistic regression analysis was conducted to assess the association between RBP4 and the presence of CKD. After adjusting for potential confounders, RBP4 was independently associated with the presence of CKD, regardless of HOMA-IR, FBG, WC, lipids, eGFR, and other common metabolic risk factors. Thirdly, the results of the ROC curve analysis showed that RBP4, as a single index (AUC: 0.666), was superior to Scr, FBG, Log HOMA-IR, WC, TG, VLDL-C, UA, HDL-C, and LDL-C. Moreover, the predictive value of RBP4, combined with routinely assessed clinical factors, was better than that of traditional risk factors. These results suggested that considering RBP4 as a potential risk factor could improve the accuracy of CKD prediction.

The correlation between RBP4 and CKD may be related to the following factors. The kidneys play an important role in the metabolism of RBP4. RBP4 usually forms a complex with transthyretin (TTR) in the blood, which helps RBP4 escape renal excretion. When RBP4 reaches the target organ, RBP4 separates from the TTR and releases retinol. Subsequently, RBP4 in the circulation is filtered by the kidney, reabsorbed, and degraded by the tubules. In addition, approximately 5% of the RBP4 in the serum circulates freely and is not bound to TTR; this portion of RBP4 is also reabsorbed and degraded by the renal tubules. Studies have shown that 99% of RBP4 is reabsorbed by the proximal renal tubules, making urinary RBP4 a highly sensitive marker of tubular dysfunction (28). In the early stages of CKD, due to the decline in glomerular filtration rate and tubular reabsorption impairment, both serum and urinary RBP levels are significantly increased. Therefore, kidney function has been proposed as a major determinant of serum RBP4 levels, which are known to deteriorate upon the onset of other metabolic diseases and thus may lead to an accumulation of RBP4 in the blood. One study tested RBP4 levels in diabetes patients with microalbuminuria and found that RBP4 concentrations were significantly elevated in the plasma of diabetes patients, with notably higher levels observed in those with microalbuminuria (29). In another study stratifying by eGFR, linear regression analysis revealed a negative influence of eGFR on RBP4 serum concentration. As eGFR decreases, RBP4 levels gradually increase (30). When renal disorders or infections cause tubular reabsorption dysfunction, serum RBP levels decrease, and urinary RBP significantly increases (31). These results indicated that kidney function is closely related to RBP4 levels. However, unlike creatinine and other kidney function markers, RBP4 indirectly affects kidney function via other pathways.

IR is a pathophysiological factor involved in CKD progression. It predisposes individuals to several metabolic disorders, such as hyperglycaemia, dyslipidaemia, and hypertension, all of which are strongly associated with poor CKD outcomes (32). Additionally, previous studies have shown that IR is an early metabolic alteration in CKD patients (33) and can occur in different stages or types of kidney disease, such as early stage CKD, Immunoglobulin A nephropathy, or polycystic kidney disease (34–36). Animal studies found that removing insulin receptors in certain kidney cells led to albuminuria and DKD-like changes, even without high blood sugar. Another study showed insulin receptor knockout in kidney cells caused albuminuria, DKD changes, and hyperglycaemia (37, 38). This suggests that IR plays a critical role in the progression of renal impairment (39). In 1997, it was reported that RBP4 was elevated in the blood of type 2 diabetes patients (39). A subsequent study found that the specific deletion of GLUT4 in adipocytes significantly increased RBP4 levels, which induced the expression of gluconeogenic enzymes in the liver and impaired insulin signalling in the muscle, thereby inducing insulin resistance. By overexpressing RBP4 in wild-type rats or injecting recombinant human RBP4, insulin resistance can be induced in rats, whereas RBP4 gene deletion can enhance insulin sensitivity (10). Berry et al. proposed another mechanism by which RBP4 affects insulin resistance, namely that RBP4 can bind to STRA6, trigger its phosphorylation, and recruit and activate Janus kinase 2 and signal transducer and activator of transcription 5, which causes the upregulation of genes such as cytokine signalling suppressor 3, thereby inhibiting insulin signalling (40). RBP4 induces insulin resistance by promoting inflammation. Mores-Vieira et al. found that antigen-presenting cells (APCs) can be activated by RBP4 through a c-Jun N-terminal protein kinase (JNK)-dependent pathway, which in turn promotes the infiltration of adipose tissue macrophages and CD4T cells, causing adipose tissue inflammation. The transfer of RBP4-activated APCs into normal mice is sufficient to induce adipose tissue inflammation, insulin resistance, and glucose intolerance (23). Another study showed that RBP4 induces the expression of pro-inflammatory cytokines in mouse and human macrophages, thereby indirectly inhibiting insulin signalling in co-cultured adipocytes. This occurs through activation of the JNK and Toll-like receptor 4 pathways, independent of the RBP4 receptor (41). Interestingly, researches indicated that RBP4 gene transcription is regulated by a multiprotein complex containing high mobility group AT-hook 1 protein (HMGA1), p54nrb/NonO, protein-associated splicing factor (PSF) and steroidogenic factor 1 (SF1)/liver receptor homologue 1 (LRH-1). HMGA1, which is involved in the regulation of steroid hormone biosynthesis, play a key role in basal and cAMP-induction of RBP4 transcription and the RBP4 and HMGA1 genes are coordinately regulated *in vitro* and *in vivo*. These studies suggest that a multi-protein complex, similar to those involved in steroid hormone gene regulation, controls RBP4 transcription. This parallels glucose metabolism-related gene regulation, implying that RBP4 expression might be part of a network linked to insulin resistance development (42, 43). Similarly, our results also found that RBP4 was positively correlated with insulin resistance, so insulin resistance played an important intermediate role in the relationship between RBP4 and CKD.

Recently, several epidemiological studies have found a positive correlation between obesity and CKD, particularly visceral obesity (VAT) (44, 45). The VAT is associated with a decline in eGFR (46, 47). Moreover, the accumulation of fat tissue at ectopic sites may cause kidney compression (48). Additionally, VAT can also induce obesity-related subclinical inflammatory changes, such as increased production of interleukin-6, tumour necrosis factor-α, and C-reactive protein, which can precede the pathogenesis of CKD (48, 49). Abnormal inflammation may lead to disturbances in kidney function and structure, such as increased albuminuria (50). Previous human studies have shown that RBP4 concentrations in the blood are related to body fat and are differentially associated with various body fat compartments. For example, one study reported that RBP4 is expressed at higher levels in visceral fat than in subcutaneous fat deposits (51). This pattern was also observed in another epidemiological study involving more than 1000 Chinese participants (52). In 102 healthy women, RBP4 concentrations were strongly and positively correlated with visceral fat mass as measured by computed tomography but not with total body fat as measured by DXA (53). Considering that computed tomography is the ‘gold standard’ for measuring visceral fat, this study confirmed the relationship between RBP4 and VAT. In our study, we used WC to measure VAT, which is also an important component of metabolic syndrome. Our results were consistent with previous ones, showing that RBP4 level was positively correlated with WC. Therefore, RBP4 may indirectly affect the kidneys by affecting VAT.

The potentially harmful effects of hyperuricaemia on CKD should not be ignored. Hyperuricaemia can increase the risk of CKD because urate crystals can be deposited in the kidneys, causing inflammation, ischaemia, fibrosis, and other damages (54). Hyperuricaemia can also lead to uric acid kidney stones, resulting in kidney obstruction and infection (55, 56). Recent studies have shown that RBP4 expression is associated with hyperuricaemia. A population-based cross-sectional survey found that RBP4 and hyperuricaemia were positively associated after adjusting for confounding factors, and the OR for the presence of hyperuricaemia compared with quartile 4 of RBP4 in quartile 1 was 7.90 (16). Chen et al. reported that elevated serum RBP4 levels were associated with increased serum uric acid levels in 885 Taiwanese individuals (57). The potential mechanisms of action of RBP4 and uric acid remain unclear, and insulin resistance factors may be involved. A recent study showed that RBP4 may be involved in hyperuricaemia-induced insulin resistance by inhibiting IRS/PI3K/Akt phosphorylation (17).

Hypertension and CKD are closely interlinked pathophysiological states; sustained hypertension can lead to poor kidney function, and a progressive decline in kidney function can conversely lead to worsened BP control. The pathophysiology of hypertension in CKD is complex and involves multiple factors, including reduced nephron mass, increased sodium retention and extracellular volume expansion, sympathetic nervous system overactivity, activation of hormones (such as the renin-angiotensin-aldosterone system), and endothelial dysfunction (58–60). Previous studies have shown that circulating RBP4 levels are associated with BP and cardiovascular disease. One study found that compared with wild-type littermates, RBP4 knockout mice had lower SBP and DBP, while RBP4 overexpressing mice had higher SBP and DBP. Moreover, RBP4-deficient mice were protected from angiotensin II-induced hypertension and cardiac hypertrophy, thereby preserving the cardiovascular system (61). In a cross-sectional study of Chinese individuals with prehypertension, RBP4 positively correlated with SBP and DBP (62). Most importantly, in 3505 healthy, CVD-free individuals participating in the Framingham Third Generation Cohort, the total RBP4 concentration was positively correlated with mean arterial pressure (63). The association between BP and RBP4 observed in this study is consistent with the above studies. Therefore, the relationship between RBP4 and CKD may involve BP-related factors.

In summary, the relationship between RBP4 and CKD involves multiple factors, including direct factors related to the breakdown and metabolism of RBP4 in the kidney and indirect factors related to the association of RBP4 with insulin resistance, diabetes, obesity, inflammation, hyperuricaemia, hypertension, and other risk factors of CKD. Interestingly, in the logistic regression model established in this study, after adjusting for common risk factors such as BP, FBG, lipids, HOMA-IR, UA, hs-CRP, age, and eGFR, RBP4 continued to exhibit significant ORs for the presence of CKD (OR: 1.862, 95%CI: 1.057-3.279) when comparing the top quartile with the bottom quartile. This suggests that RBP4 may have other potential mechanisms that affect kidney function. One animal study reported that RBP4 promotes mitochondrial dysfunction in the liver, leading to intracellular fat accumulation and increased triglyceride levels (64). An important link exists between CKD and NAFLD, and liver abnormalities can affect CKD through lipoprotein metabolism and hepatokine secretion (65). In addition, there is still intense debate about the relationship between RBP4 and IR. Although most studies and many independent laboratories have confirmed that circulating RBP4 levels are elevated in IR and type 2 diabetic states, the underlying cause of this elevation and whether RBP4 actively promotes insulin resistance remain unclear (66). Not all studies have found increased RBP4 expression in the adipose tissue of obese patients (67), and some studies have failed to reproduce the improvement in insulin sensitivity and glucose tolerance in RBP4-deficient mice fed a high-fat diet (68). Moreover, by acute or chronic liver-specific overexpression of mouse RBP4 (rather than human protein), circulating RBP4 levels increased to a level comparable to that in insulin-resistant states, but no impairment of the insulin response and glucose tolerance was observed (69, 70). In conclusion, RBP4 is an important novel adipokine associated with many metabolic diseases; however, its potential mechanisms and effects have not been fully elucidated. More studies are needed to address these issues to better elucidate the link between RBP4 and CKD. But at least for now, considering RBP4 as a potential risk factor could improve the accuracy of CKD prediction.

This study had several limitations. Firstly, it did not use a hyperinsulinaemic-euglycemic glucose clamp to measure insulin resistance, which is the ‘gold standard’ for measuring insulin sensitivity. The use of alternative methods, such as HOMA-IR, may deviate from the actual situation. Additionally, the study did not measure Hemoglobin A1c (HbA1c) levels, which may introduce a certain degree of bias in the diagnosis of the diabetic population. Secondly, this study did not fully adjust for confounding factors related to RBP4, such as liver factors. The relationship between RBP4 and non-alcoholic fatty liver disease has also been reported in the literature, and there is a cross-link between the liver and the kidney; therefore, non-alcoholic fatty liver disease is also a risk factor for CKD, but this study did not include this factor owing to the lack of relevant measurement data. Finally, all the participants were Han Chinese adults from Zhuhai City, and the results may not be generalisable to other ethnic groups. In addition, this was a single-centre study and, therefore, inevitably limited in terms of sample size. Large multicentre studies are warranted to verify these conclusions.

## Data Availability

The raw data supporting the conclusions of this article will be made available by the authors, without undue reservation.
